# Identifying functionally relevant candidate genes for inflexible ethanol intake in mice and humans using a guilt‐by‐association approach

**DOI:** 10.1002/brb3.1879

**Published:** 2020-10-23

**Authors:** Luana Martins de Carvalho, Pablo A. S. Fonseca, Isadora M. Paiva, Samara Damasceno, Agatha S. B. Pedersen, Daniel da Silva e Silva, Corinde E. Wiers, Nora D. Volkow, Ana L. Brunialti Godard

**Affiliations:** ^1^ Laboratório de Genética Animal e Humana Departamento de Genética Universidade Federal de Minas Gerais (UFMG) Belo Horizonte Brazil; ^2^ Laboratory of Neuroimaging National Institute on Alcohol Abuse and Alcoholism National Institutes of Health Bethesda USA; ^3^ Center for Alcohol Research in Epigenetics Department of Psychiatry University of Illinois at Chicago Chicago IL USA; ^4^ Laboratório de Genética Humana e Médica Departamento de Genética Universidade Federal de Minas Gerais (UFMG) Belo Horizonte Brazil; ^5^ University of Guelph Department of Animal Biosciences Centre for Genetic Improvement of Livestock Guelph Ontario Canada; ^6^ Laboratory on the Neurobiology of Compulsive Behavior National Institute on Alcohol Abuse and Alcoholism National Institutes of Health Bethesda MD USA; ^7^ National Institute on Drug Abuse Bethesda, National Institute of Health Bethesda MD USA

**Keywords:** alcohol use disorders, GUILDify, IRF4, LRRK, microarray data, prefrontal cortex, striatum, ToppGene OR guilt‐by‐association approaches

## Abstract

Gene prioritization approaches are useful tools to explore and select candidate genes in transcriptome studies. Knowing the importance of processes such as neuronal activity, intracellular signal transduction, and synapse plasticity to the development and maintenance of compulsive ethanol drinking, the aim of the present study was to explore and identify functional candidate genes associated with these processes in an animal model of inflexible pattern of ethanol intake. To do this, we applied a guilt‐by‐association approach, using the GUILDify and ToppGene software, in our previously published microarray data from the prefrontal cortex (PFC) and striatum of inflexible drinker mice. We then tested some of the prioritized genes that showed a tissue‐specific pattern in postmortem brain tissue (PFC and nucleus accumbens (NAc)) from humans with alcohol use disorder (AUD). In the mouse brain, we prioritized 44 genes in PFC and 26 in striatum, which showed opposite regulation patterns in PFC and striatum. The most prioritized of them (i.e., *Plcb1 and Prkcb* in PFC, and *Dnm2* and *Lrrk2* in striatum) were associated with synaptic neuroplasticity, a neuroadaptation associated with excessive ethanol drinking. The identification of transcription factors among the prioritized genes suggests a crucial role for *Irf4* in the pattern of regulation observed between PFC and striatum. Lastly, the differential transcription of *IRF4* and *LRRK2* in PFC and nucleus accumbens in postmortem brains from AUD compared to control highlights their involvement in compulsive ethanol drinking in humans and mice.

## Graphical abstract

Gene prioritization approaches are useful tools to explore and select candidate genes in transcriptome studies. To identify functional candidate genes associated with these processes in an animal model of inflexible pattern of ethanol intake we applied a guilt‐by‐association approach using the GUILDify and ToppGene software. Identification of transcription factors among the prioritized genes suggests a crucial role for Irf4 in the pattern of regulation observed between PFC and striatum.

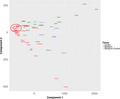

## 1 INTRODUCTION

Alcoholism is a chronic disorder characterized by compulsive ethanol seeking and intake despite negative consequences (Koob & Volkow, 2016). Among the brain regions altered by chronic ethanol intake, the striatum and the prefrontal cortex (PFC) are considered central to reinforcement and decision‐making over ethanol consumption (Koob & Volkow, 2010). Recently, studies have been showing the different pattern of gene expression among brain regions in both human and animal models of chronic alcohol administration (Bogenpohl et al., 2019; Farris et al., 2015). It is proposed that changes in the regulation of gene expression contribute to the long‐lasting changes in chronic ethanol‐induced neuronal plasticity resulting in inflexible changes in behavior (Nestler, 2001).

We published two transcriptional studies, in the PFC and striatum, that compared *inflexible drinker* mice (consume ethanol despite negative consequences, akin to human alcohol addiction) to *light drinker* mice (mice who preferred water before and after withdrawal and after ethanol adulteration with quinine) (da Silva E de Paiva Lima et al., 2017; Silva et al., 2016). The transcriptional analysis revealed that the *Lrrk2*, *Camk2a*, *Camk2n1*, *Pkp2,* and *Gja1* genes were differentially regulated in *inflexible drinkers* compared to *light drinkers*, implicating them in the loss of control over ethanol consumption (da Silva E de Paiva Lima et al., 2017; Silva et al., 2016). However, there were many other genes differentially expressed in PFC and striatum that remained unexplored. In this regard, gene prioritization approach emerges as an extremely useful tool to explore and select remaining candidate genes from transcriptome studies that could be associated with a specific disease or condition (Albert & Lemonde, 2004; Kominakis et al., 2017; Tian et al., 2008). The “guilt‐by‐association approach” is one type of network‐based prioritization tools, which principle suggests that the genes whose products (proteins) interact with the products of known disease genes are more likely to be disease genes (Guney et al., 2014).

The goal of the present study was to identify functional candidate genes associated with the regulation of dopamine pathways, neuronal activity, intracellular signal transduction, synapse plasticity, and behaviors, due to the relevance of these processes for the control of ethanol intake. To explore the genes on microarrays and identify possible candidate genes, we used a guilt‐by‐association approach that took into consideration the ethanol‐induced neurobiological process already described in the literature. We then tested some of the prioritized genes that showed a tissue‐specific pattern in postmortem brain tissue (PFC and nucleus accumbens (NAc)) from humans with alcohol use disorder (AUD). This preclinical postmortem translational study allowed us to corroborate functional candidate genes in PFC and striatum of an animal model of inflexible drinking to that from expression patterns in postmortem brain samples of individuals with AUD.

## 2 METHODS

### 2.1 Extended chronic ethanol intake

The present study was performed with striatum and PFC microarray data previously published by our group (da Silva E de Paiva Lima et al., 2017; Silva et al., 2016). These studies used samples from the animal model reported by Ribeiro and colleagues (Ribeiro et al., 2012), and a detailed description of experimental design, ethanol consumption, and blood ethanol concentration is published in (Ribeiro et al., 2012).

In short, Swiss male mice were subjected to a three‐bottle free‐choice treatment: a 10% and a 5% (v/v) ethanol solution, and water. Only male mice were used to avoid interference of hormonal fluctuation, since we know that estrogen can enhance the reinforcing and rewarding effects of alcohol, contributing to the increase of alcohol intake in female mice (Hilderbrand & Lasek, 2018; Vandegrift et al., 2017). The experimental design consisted of four steps: (1) acquisition/free‐choice (AC: 10 weeks) with simultaneous access to water and ethanol solutions 5 and 10% (v/v); (2) withdrawal of ethanol solutions (2 weeks); (3) reinstatement of ethanol solutions (RE: 2 weeks); and (4) adulteration of ethanol solutions with 0.005 g/L quinine (AD: 2 weeks). The control group had access to water only throughout the experiment (Ribeiro et al., 2012). The Swiss mice are an outbred strain and were chosen for this model in order to accesses the phenotypic variability in the pattern of alcohol intake that could reflect the genotypic variability, thus representing better what is observed in the human.

At the end of the three‐bottle free‐choice paradigm, mice were classified based on their ethanol consumption and preference: “light drinkers” (significant higher water than ethanol consumption throughout all experiment phases); “heavy drinkers” (higher ethanol consumption than water with significant reduction of ethanol intake after adulteration with quinine); and “inflexible drinkers” (higher ethanol than water consumption throughout the experiment, without significant reduction in ethanol intake after adulteration with quinine). The individual ethanol consumption profile is shown in Table S1. Animals that did not meet any of the classification criteria were excluded. At the end of the AD phase, mice from Inflexible, Heavy, and Light groups were exposed to the same free‐choice task for an extra week, allowing them to return to their previous ethanol intake patterns.

Inflexible drinkers showed high and stable ethanol consumption even under an aversive condition generated by quinine, a nonpalatable compound. Quinine adulteration is a well‐established approach that can be used to model compulsive drinking in animals and is a suitable mode to demonstrate aversion resistance that has face validity for human alcoholism (Blegen et al., 2018; H. Chen & Lasek, 2019; Hopf & Lesscher, 2014). In addition, this group (113 ± 11.3 mg/dl) along with the heavy drinker mice (79 ± 19.8 mg/dl) presented intoxication levels of BEC that were significantly higher than those in the light drinkers (48 ± 13.3 mg/dl) (Ribeiro et al., 2012).

### 2.2 Microarray analysis

The gene expression of bilateral striatum (dorsal and ventral) and PFC was analyzed using an Affymetrix GeneChip® Mouse Genome 430 2.0 Array (Affymetrix, São Paulo, Brazil). For light and inflexible drinkers, a pooled sample of 4 animals of each group was hybridized in triplicates, totalizing 6 chips. The evaluation of the two extreme ethanol drinking groups allowed us to find possible genes related to the compulsive drinking phenotype while controlling for the chronic presence of alcohol even though BEC levels differed (da Silva E de Paiva Lima et al., 2017; Silva et al., 2016). The fragmentation and hybridization steps were performed in accordance with GeneChip 3’IVT Express Kit (Affymetrix, São Paulo, Brazil) manual. The fluorescent scanning step was performed using the GeneChip® Scanner 3,000 (Affymetrix, São Paulo, Brazil). The array data were normalized using the RMA (Robust Multi‐array Average) method using the package “affy” in the R environment. Differentially expressed genes (DEGs) were identified using the RankProd algorithm with a significance level set at 99% (*p* < .01). After the acquisition of DEG list, the R package *mouse4302.db* (version 3.2.2) was used to retrieve important information such as gene name, chromosome loci, and function for each array probe. The volcano plot and heatmap clustering analysis showing the differentially expressed genes can be found in (da Silva E Silva et al., 2016). The microarray data are available on the Gene Expression Omnibus (GEO), NCBI, and can be assessed using the following ID: GSE123114.

For the present study, we used the DEG list generated by the analysis described above. As we choose to apply a guilt‐by‐association approach to prioritize those genes, we considered the module fold change (FC) values >1.3 to do the first gene selection. This value reflects at least a 30% expression difference (either for up or downregulated) in the Inflexible drinkers versus light drinkers, given us a higher number of genes to start the analysis. The value of >1.3 has been used by others [17, 18,19], and it is an effect size large enough to assess the relationship between two variables and determine their biological relevance.

### 2.3 Functional prioritization of differentially expressed genes

**Figure 1:**
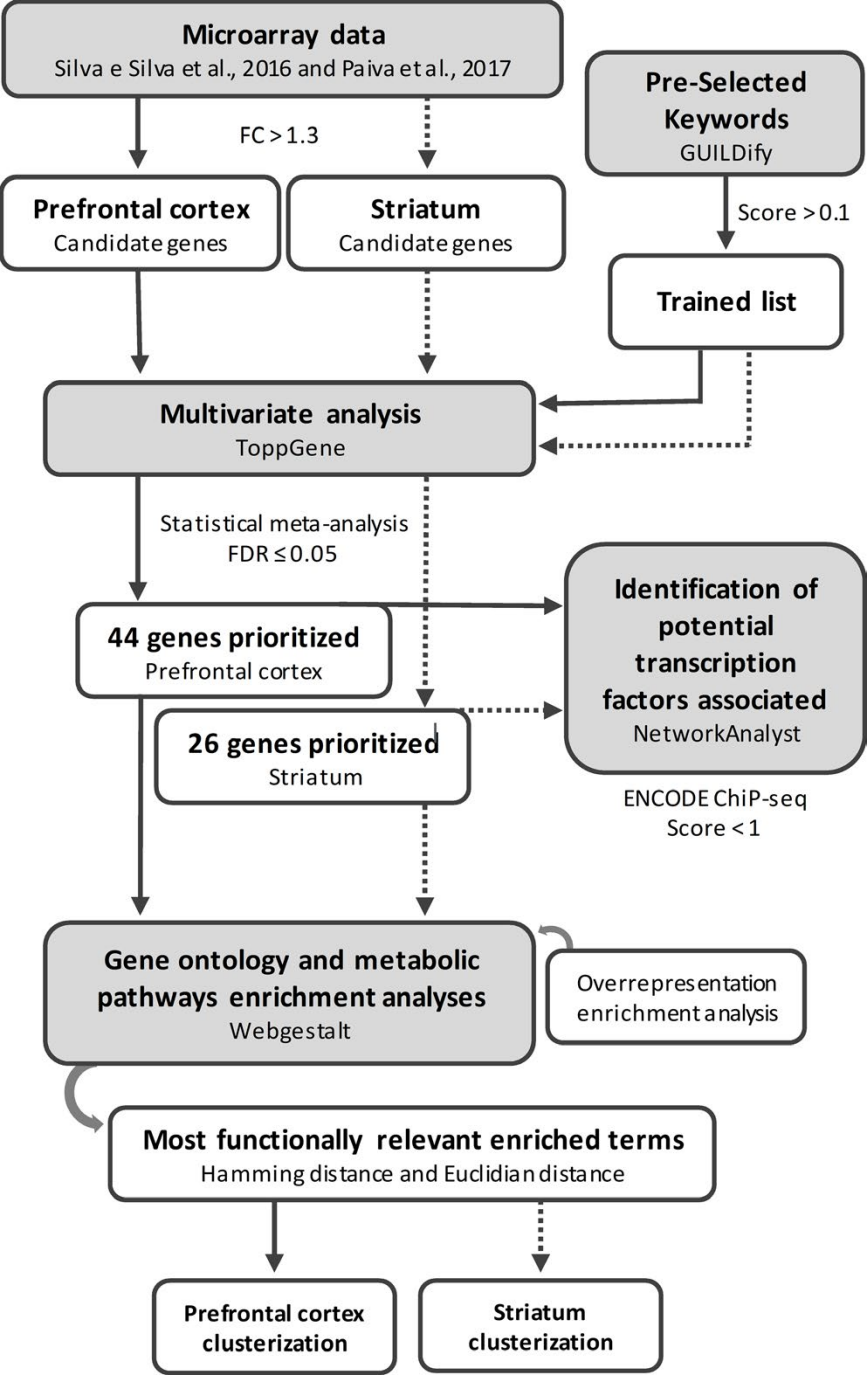
Workflow of gene prioritization on the microarrays analysis in Prefrontal cortex (PFC) and striatum of inflexible drinker mice. Using keywords that describe the biological process that underlies addiction and compulsive ethanol intake, GUILDify generated a list of genes associated with these phenotypes (trained list). ToppGene related the functional information of the trained list genes with candidate genes of the microarray of each structure separately through a fuzzy‐based multivariate analysis, which generated a list of prioritized genes. WebGestalt was used to perform Gene Ontology and metabolic pathway enrichment analyses for the prioritized genes in PFC and striatum with assistance of an overrepresentation enrichment analysis. Subsequently, the most functional relevant enriched terms were selected and the Hamming distance among the genes was estimated using an incidence matrix composed of the genes and the terms. Subsequently, the Hamming distance was used to calculate the Euclidian distance and the prioritized genes were clustered. Finally, NetworkAnalyst was used to identify potential transcription factors with higher regulatory potential for the prioritized genes in PFC and striatum. The microarray data are available on the Gene Expression Omnibus (GEO), NCBI, and can be assessed using the following ID: GSE123114

The prioritization of DEG followed three steps: (1) DEG in the microarray analysis for PFC and striatum were selected based on fold change value to generate the statistical candidate gene list; (2) GUILDify software was used to retrieve well‐established functional candidate genes (trained list) for the neurobiological process already known to be triggered by alcohol to induces its effects, through keywords selection; and (3) ToppGene software was used to perform a candidate gene prioritization using simultaneously the trained list and the statistical candidate gene list. The workflow is represented in Figure 1.

In the first step, DEGs from the microarray data for PFC and Striatum (da Silva E de Paiva Lima et al., 2017; Silva et al., 2016) were selected based on FC > 1.3, to generate the statistical candidate gene list. In the second step, the GUILDify database (BIANA knowledge base) was used to link genes and phenotypes in animal models (*Mus musculus*). GUILDify uses keywords chosen by users to search in UniProt, OMIM, and GO databases, and products of genes (proteins) that match these keywords. GUILDify maps the selected proteins onto a genome‐wide protein interaction network (PPI) and runs the global topology‐based prioritization algorithm (NetScore). As an output, GUILDify provides a likelihood score (GUILDify score) associating the gene product with the phenotype for each gene product in the PPI network (Guney et al., 2014).

To obtain the “trained list” of candidate genes, we used keywords that describe neurobiological process already known to be induced by ethanol such as alterations in intracellular signal transduction, neuronal activity, and inflexible changes in behavior. Together, all these processes contribute to the long‐lasting changes in chronic ethanol‐induced neuronal plasticity and consequently to the perpetuation of addiction cycle. Consequently, using the guilt‐by‐association approach, we aimed to identify putative functional candidate genes for these relevant processes. The selected keywords were as follows: "Nicotinic acetylcholine", "Inhibits ionotropic glutamate", "Inhibition NMDA", "BK", "GIRK", "SK2", "Mesolimbic dopamine", "Firing midbrain dopamine", "Dopamine neuron fire", "dopamine", "kappa opiate", "striatal D2 dopamine", "midbrain GABAergic", "GABAA potentiation", "Glutamatergic transmission", "Glutamate reuptake", "Long‐term depression", "Long ‐term potentiation", "Ethanol‐binding", "Ethanol‐sensitive", "ethanol potentiation", "Long‐term ethanol exposure", "motor impulsivity", "Ethanol‐receptor", "Ethanol‐associated behaviors", "Large conductance Ca2+‐activated K+", "addiction" "compulsion", "craving". Only those genes with a GUILDify score >0.1 were retained in the final “trained list” (Table S2).

In the third step, ToppGene was used to perform a candidate gene prioritization using the trained list (obtained with GUILDify) and the candidate gene list (FC > 1.3) simultaneously. Briefly, ToppGene performs an annotation‐based prioritization analysis through a fuzzy‐based multivariate approach to compute the similarity between any two genes based on semantic annotations. The similarity scores from individual features are combined into an overall score using a statistical meta‐analysis. A p‐value of each annotation of a test gene is derived by random sampling of the whole genome (J. Chen et al., 2009).

In our study, the functional information shared between the “trained” gene list and the candidate genes was used to perform the multivariate analysis. The following sources were used to retrieve the functional information for the genes in both lists: Gene Ontology (GO) terms for molecular function (MF), biological process (BP), and cellular component (CC); human and mouse phenotypes; metabolic pathways; PubMed publications; coexpression pattern; and diseases. Finally, *p*‐values were obtained using a statistical meta‐analysis, where a random sampling of 5,000 genes from the whole genome for each annotation information was combined to estimate an overall p‐value. Subsequently, a false discovery rate (FDR) of 5% multiple correction (*p*‐value ≤ 10e‐4) was applied and the significant prioritized genes were selected. It is important to highlight that those genes that were present in both the trained and candidate gene lists were automatically selected as prioritized genes. These analyses were performed independently for the candidate genes identified in striatum and PFC.

### 2.4 Gene Ontology and metabolic pathway enrichment analyses

The WebGestalt application was used to perform the Gene Ontology (GO) and metabolic pathway enrichment analyses for the prioritized genes in striatum and PFC, independently (Zhang et al., 2005). An overrepresentation enrichment analysis (ORA) was performed for each GO term category (biological process (BP), molecular function (MF), and cellular component (CC)) using a nonredundant database. The ORA was also performed for the metabolic pathways present in the Kyoto Encyclopedia of Genes and Genomes (KEGG). For both analyses, the terms analyzed were annotated specifically for the *Mus musculus* genome. Terms were considered enriched with a *p*‐value < .05 and FDR 5% multiple correction testing and visualization of results was performed using the GOplot package on R statistical software (R Core Team, 2013; Walter et al., 2015). These analyses were performed independently for the candidate genes identified in striatum and PFC. To evaluate the fold change profile in each enriched term, a z‐score was calculated using the following formula:

$$z‐score=\frac{\left(up‐down\right)}{\mathrm{count}}$$

where up is the number of genes with positive fold change, down is the number of genes with negative fold change, and count is the total number of genes related to the enriched term.

To evaluate the functional similarity of the prioritized genes, the enriched terms associated with the selected processes used during the guilty‐by‐association approach were selected and the hamming distance among the genes was estimated using the incidence matrix composed by the genes and the enriched terms. The hamming distance matrix was used to compute the number of differences among the DEG regarding the enriched terms (obtained from BP, MF, CC, and KEGG terms). In other words, all the pairs of DEG were compared using an incidence matrix to account the number of enriched processes were annotated to the first gene of the pair but not the second gene from the pair and vice versa. Consequently, the hamming distance matrix, obtained from the original incidence matrix (composed by genes and enriched terms), was used to calculate the Euclidian distance between the pairs of genes, resulting in a similarity matrix. Once the Euclidean distance was calculated, the similarity matrix was used as input of the multidimensional scaling analysis in order to create a map of the distances among the genes using two dimensions. Subsequently, the proportion of the variance explained by the two dimensions used to create the distance map among the genes was calculated.

### 2.5 Identification of potential transcription factor for the best functional candidate genes

The prioritized genes identified in the striatum and PFC were subjected to a gene network analysis in order to identify potential transcription factors (TFs) using the NetworkAnalyst application (Xia et al., 2015). The potential TFs were obtained from the ENCODE ChIP‐seq data using only peak intensity signal <500 and the predicted regulatory potential score <1 (using BETA Minus algorithm). Subsequently, a “regulatory network” was created using the interactions between the prioritized genes and the potential TFs, where the nodes represent either the genes or the TF (circles and squares, respectively), and the edges represent the predicted interaction between them. The centrality metrics (degree and betweenness) for each network were analyzed to identify those TFs that explain most of the network topology. Consequently, using this methodology, it is possible to identify those TFs that have a higher regulatory potential for the functionally prioritized genes. To evaluate the relationship between the potential TFs and the prioritized genes between and within tissues, we used a Venn diagram.

### 2.6 Postmortem Human Brain: subjects, clinical assessment, behavioral measures, and real‐time PCR

Human postmortem brain tissue was obtained from the New South Wales Tissue Resource Centre (NSWBTRC) at the University of Sydney, Australia. PFC and nucleus accumbens (NAc) were analyzed from males with severe AUD (PFC: *n* = 10 and NAc: *n* = 8) and from male controls (PFC: *n* = 13 and NAc: *n* = 12) that consumed less than 20 g of absolute alcohol per day (Sutherland et al., 2016). All AUD subjects had alcohol detected in blood at the time of death. The numbers for the brain regions differed due to tissue availability.

Clinical characteristics of AUD and control subjects were retrospectively assessed through extensive review of all available medical files followed by a confirmation through donor history questionnaires from the donor's next of kin. Clinical characterization of alcohol use was based on Diagnostic Criteria for Alcohol‐Related Disorders‐Alcohol Dependence (DSM‐IV). Alcohol use disorders identification test (AUDIT) was used to assess alcohol consumption, drinking behaviors, and alcohol‐related problems. The number of standard drinks per week and per day was calculated based on an Australian standard drink that contains 10 grams of alcohol. Quantity and frequency of smoking were also retrospectively assessed, and pack‐years of smoking were calculated. All details about how NSWBTRC collects demographic, social, medical, pathological, cognitive, psychiatric medication and lifestyle factor data are published in (Sutherland et al., 2016).

Total mRNA was extracted from PFC and NAc, (superior frontal Brodmann areas 8 and 9) using the RNeasy Lipid Tissue Mini Kit (Qiagen) in accordance with the manufacturer's instructions. Samples were quantified using an Agilent 2,100 Bioanalyzer and an RNA 6,000 Nano Kit and stored at −80°C. For each sample, 1 μg of total RNA was used to make complementary DNA (cDNA) using SuperScript® III First‐Strand Synthesis SuperMix for qRT‐PCR kit (Invitrogen) in accordance with the manufacturer's instructions.

The expression levels of target genes were measured using ViiA™ 7 Real‐Time PCR System (Thermo Fisher). The following TaqMan Gene Expression Assays were used: leucine‐rich repeat kinase 2 (*LRRK2*) Hs01115057_m1, interferon regulatory factor 4 (*IRF4*) Hs00180031_m1, dynamin 2 (*DNM2*) Hs00974698_m1, protein kinase C beta (*PRKCB*) Hs00176998_m1, and phospholipase C beta 1 (*PLCB1*) Hs01001930_m1.

Real‐time PCR reactions for each gene were performed using 10 µl of TaqMan™ Universal PCR Master Mix (Thermo Fisher), 0.5 µl of TaqMan assay, and 3.5 µl of ultra‐pure water. For all reactions, a negative control without cDNA template (NTC) was tested, and the final reaction volume was kept at 10 µl. The relative quantities of the transcripts were calculated by the delta–delta Ct method (Pfaffl, 2001) using the GADPH gene as a endogenous control according to Vandesompele et al. (2002). Data were analyzed for the Gaussian distribution using the Shapiro–Wilk and Anderson–Darling normality tests. ROUT method was used to identify outliers (Q = 1%). Independent *t* tests were used to calculate differences in gene expression between AUD and controls for IRF4 and DNM2 in NAc and for IRF4 and PRKCB in PFC. The Mann–Whitney test was used for LRRK2 in NAc and PLCB1 in PFC. We report both uncorrected (*p* < .05) and corrected false discovery rate 5% (FDR) corrected (described as q value) results. Statistical tests were performed using GraphPad Prism version 7.01 and R software.

### 2.7 Ethics statement

Animal experimentation was carried out in compliance with institutional guidelines and approved by the Ethics Committee for Animal Experimentation of the Universidade Federal de Minas Gerais (protocol number 159/2007) and the Universidade Federal do Paraná (Protocol Number: 281) (Ribeiro et al., 2012).

The use of human postmortem brain tissue was reviewed and approved by a National Institute on Alcohol Abuse and Alcoholism (NIAAA) Scientific Advisory Board, and the project was also reviewed by the National Institutes of Health (NIH) Office of Human Subjects Research Protections and determined exempt from review by the NIH Institutional Review Board.

## 3 RESULTS

### 3.1 Prioritized genes

Analyses started with 1674 and 917 DEGs in PFC and striatum, respectively, obtained from the microarray data (da Silva E de Paiva Lima et al., 2017; Silva et al., 2016). The microarray data are available on the Gene Expression Omnibus (GEO), NCBI, and can be assessed using the following ID: GSE123114. After screening for the threshold > 1.3‐fold change, 1,550 and 820 DE genes from PFC and striatum, respectively, were selected, giving rise to the statistical candidate gene list. In the second step, GUILDify generated a trained list with 3,946 genes associated with the preselected keywords. At the final step, 44 and 26 functional candidate genes (p‐value after FDR 5%<0.05), in PFC and striatum, respectively (Table 1 and Table 2 and Table S4), were selected in the functional prioritization analysis performed by the ToppGene software, using both the statistical candidate gene list and the trained list. Interestingly, 10 prioritized genes were shared between both tissues, where 5 genes (*Atp1a3, Camk2a, Dnm1, Gabrb3,* and *Gria1*) were directly selected from the overlapping between the statistical candidate gene list and trained list obtained with GUILDify and the other 5 (*Kcnma1, Lct, Meis2, Palm, and Slc17a7*) were selected through the prioritization performed by ToppGene software.

**Table 1:** Prioritized genes in prefrontal cortex. ToppGene related the functional information (retrieved from Gene Ontology; PubMed publications; coexpression pattern; and diseases) of the trained list genes with candidate genes of the microarray of each structure separately through a fuzzy‐based multivariate analyze, which generated a list of prioritized genes. *Genes were prioritized in both prefrontal cortex and striatum. The microarray data are available on the Gene Expression Omnibus (GEO), NCBI, and can be assessed using the following ID: GSE123114

Prefrontal Cortex			
Gene Symbol	Gene ID	Description	*p*‐value
*Anxa1*	301	Annexin A1	1,44E−04
*Apc*	324	APC regulator of WNT signaling pathway	5,21E−05
*Atp1a3	478	Atpase Na+/K + transporting subunit alpha 3	5,67E−05
*B2m*	567	Beta−2 microglobulin	1,18E−04
*Cacna1g*	8,913	Calcium voltage‐gated channel subunit alpha1 G	1,68E−04
*Camk2a	815	Calcium/calmodulin‐dependent protein kinase II alpha	7,83E−05
*Camk2b*	816	Calcium/calmodulin‐dependent protein kinase II, beta	1,28E−04
*Cask*	8,573	Calcium/calmodulin‐dependent serine protein kinase	1,74E−04
*Cdh1*	999	Cadherin 1	6,79E−05
*Cdkn1a*	1,026	Cyclin‐dependent kinase inhibitor 1A	1,72E−04
*Cxcl12*	6,387	Chemokine ligand 12	4,12E−05
*Ddc*	1644	Dopa decarboxylase	2,29E−04
*Dnm1	1759	Dynamin 1	6,97E−05
*Drd2*	1813	Dopamine receptor D2	1,16E−04
*Erbb3*	2065	Erb‐b2 receptor tyrosine kinase 3	4,43E−05
*Fn1*	2,335	Fibronectin 1	5,25E−05
*Fos*	2,353	FBJ osteosarcoma oncogene	8,11E−05
*Gabra2*	2,555	GABA A receptor, subunit alpha 2	1,85E−04
*Gabrb3	2,562	GABA A receptor, subunit beta 3	4,30E−05
*Gnai2*	2,771	G‐protein subunit alpha i2	1,47E−04
*Gria1	2,890	Glutamate ionotropic receptor AMPA type subunit 1	9,54E−05
*Hla‐Dqb1*	3,119	Major histocompatibility complex, class II, DQ beta 1	3,15E−05
*Igf1*	3,479	Insulin‐like growth factor 1	6,79E−05
*Jun*	3,725	Jun proto‐oncogene	1,23E−04
*Kcnq2*	3,785	Potassium voltage‐gated channel subfamily Q member 2	8,24E−05
*Kit*	3,815	KIT proto‐oncogene receptor tyrosine kinase	2,45E−05
*Limk1*	3,984	LIM domain‐containing, protein kinase	1,40E−04
*Lrp1*	4,035	Low‐density lipoprotein receptor‐related protein 1	2,19E−04
*Mapt*	4,137	Microtubule‐associated protein tau	5,15E−07
*Mef2c*	4,208	Myocyte enhancer factor 2C	8,03E−05
*Nrp1*	8,829	Neuropilin 1	2,19E−04
*Pdgfb*	5,155	Platelet derived growth factor, B polypeptide	3,17E−05
*Plcb1*	23,236	Phospholipase C, beta 1	7,44E−05
*Plcb4*	5,332	Phospholipase C, beta 4	1,24E−04
*Prkar1b*	5,575	Protein kinase, camp‐dependent regulatory, type I beta	2,15E−04
*Prkcd*	5,580	Protein kinase C, delta	9,55E−05
*Slc1a1*	6,505	Solute carrier family, member 1	2,19E−04
*Stat1*	6,772	Signal transducer and activator of transcription 1	1,88E−04
*Stx1a*	6,804	Syntaxin 1A	7,43E−05
*Tgfb2*	7,042	Transforming growth factor, beta 2	6,95E−05
*Tgfb3*	7,043	Transforming growth factor, beta 3	1,73E−04
*Th*	7,054	Tyrosine hydroxylase	7,06E−05
*Wnt5a*	7,474	Wnt family member 5A	1,55E−04
*Ywhaz*	7,534	Tyrosine 3‐monooxygenase/tryptophan 5‐monooxygenase activation protein zeta	1,29E−04

**Table 2:** Prioritized genes in striatum. ToppGene related the functional information (retrieved from Gene Ontology; PubMed publications; coexpression pattern; and diseases) of the trained list genes with candidate genes of the microarray of each structure separately through a fuzzy‐based multivariate analysis, which generated a list of prioritized genes. *Genes were prioritized in both prefrontal cortex and striatum. The microarray data are available on the Gene Expression Omnibus (GEO), NCBI, and can be assessed using the following ID: GSE123114

Striatum			
Gene Symbol	Gene ID	Description	p‐value
*Atp1a3	478	Atpase Na+/K + transporting subunit alpha 3	1,83E−05
*Atxn2*	6,311	Ataxin 2	4,33E−05
*Bmpr2*	659	Bone morphogenetic protein receptor, type II	1,88E−04
*Braf*	673	Braf transforming gene	4,08E−05
*Cacna2d1*	781	Calcium channel, voltage‐dependent, alpha2/delta subunit 1	1,88E−04
*Camk2a	815	Calcium/calmodulin‐dependent protein kinase II alpha	3,83E−05
*Cdkn1b*	1,027	Cyclin‐dependent kinase inhibitor 1B	1,06E−04
*Dnm1	1759	Dynamin 1	3,08E−05
*Dnm2*	1785	Dynamin 2	6,12E−05
*Drd2*	1813	Dopamine receptor D2	4,73E−05
*Gabrb3	2,562	GABA A receptor, subunit beta 3	1,34E−05
*Gria1	2,890	Glutamate receptor, ionotropic, AMPA1 (alpha 1)	2,80E−05
*Hbegf*	1839	Heparin‐binding EGF‐like growth factor	1,86E−04
*Hspa8*	3,312	Heat shock protein 8	1,99E−04
*Irf4*	3,662	Interferon regulatory factor 4	7,62E−05
*Kalrn*	8,997	Kalirin, rhogef kinase	2,20E−04
*Kcnj6*	3,763	Potassium inwardly rectifying channel subfamily J member 6	1,00E−04
*Kif1b*	23,095	Kinesin family member 1B	2,12E−04
*Mapk8*	5,599	Mitogen‐activated protein kinase 8	2,13E−04
*Mef2c*	4,208	Myocyte enhancer factor 2C	1,39E−04
*Pafah1b1*	5,048	Platelet‐activating factor acetylhydrolase, isoform 1b, subunit 1	1,11E−04
*Prkar2b*	5,577	Protein kinase, camp‐dependent regulatory, type II beta	1,08E−04
*Scn1a*	6,323	Sodium channel, voltage‐gated, type I, alpha	7,14E−05
*Scn1b*	6,324	Sodium channel, voltage‐gated, type I, beta	8,34E−05
*Slc1a2*	6,506	Solute carrier family 1, member 2	1,18E−04
*Syn1*	6,853	Synapsin I	1,94E−04
			

### 3.2 Enrichment analyses for prioritized genes

**Figure 2:**
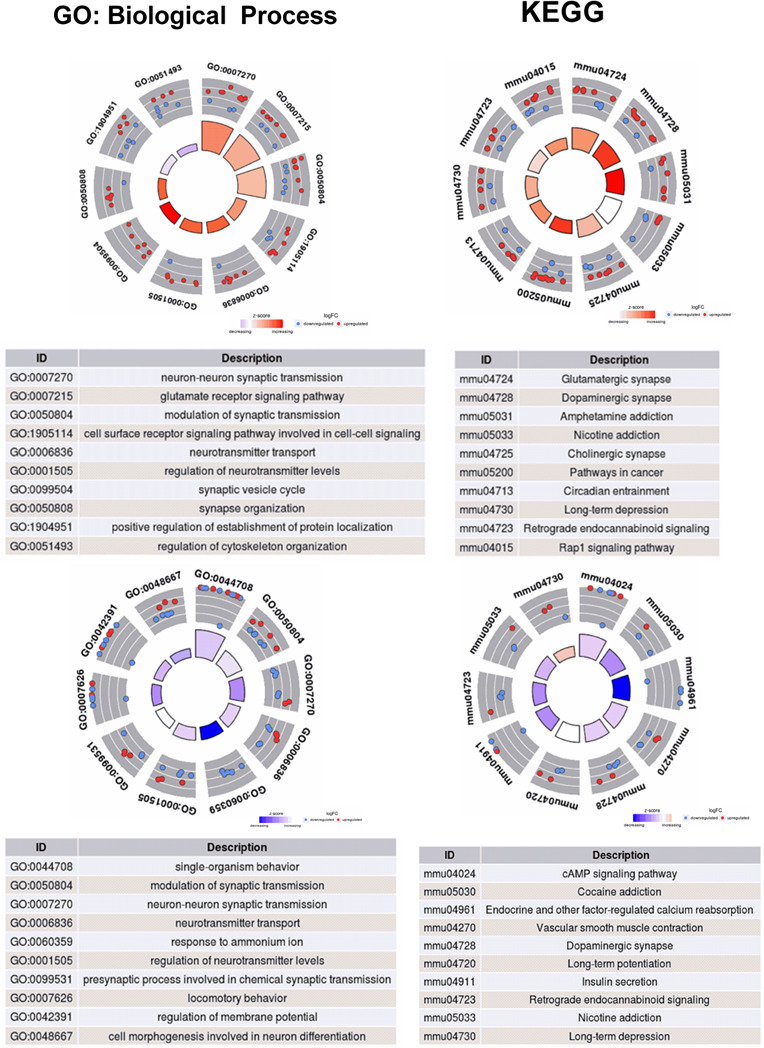
Circle plots for the most functionally relevant enriched terms for PFC (first row) and striatum (second row), depicting the relationship between the enriched terms and the gene expression profile for biological processes (first column) and KEGG pathways (second column). The outer circle indicates the up‐ (red dots) or downregulate (blue dots) state of each gene associated with each term. The inner circle represents the z‐score calculated for each term using the number of up‐ and downregulated genes. Negative z‐scores indicate a downregulation of the genes annotated for the current biological process or KEGG pathways. Positive z‐scores indicate upregulation of the genes annotated for the current biological process or KEGG pathways. For the biological process enriched terms in PFC and striatum, only the 10 most significant terms were shown in order to keep all the IDs legible

Figure 2 shows the enrichment analyses for the prioritized genes in PFC and striatum, independently. Circle plots represent the enriched terms associated with the candidate processes used during the guilty‐by‐association analysis for PFC and striatum, indicating the relationship between the enriched terms and the gene expression profile for BP and KEGG pathways. To facilitate the visualization and reduce the “noise” in the enrichment analyses, the most functional relevant terms were depicted in the barplot for the enriched GO and KEGG terms (see Table S3). Several terms related to the regulation of the nervous system (i.e., synaptic transmission, synaptic vesicle cycle, regulation of membrane potential), behavior, and response to stimulus were identified as enriched in both functional candidate genes’ list. These results reinforce the potential of guilt‐by‐association approaches to identify candidate genes associated with target phenotypes among a new list of candidate genes using the functional profile of previously reported candidate genes. Tables S5 and S6 present all the enriched terms for GO and KEGG analyses. Figure S1 presents the classification outcomes for molecular functions and cellular components obtained with GO.

The fold change profile, accessed by the z‐score of up‐ and downregulated prioritized genes in each enriched term, provided the following pattern between PFC and striatum: in almost all the cases, the enriched terms in the PFC were composed by a set of upregulated genes, while in striatum they were composed mostly by a different set of downregulated genes (Figure 2).

Interestingly, when the z‐score is calculated for all the DEGs and the prioritized genes in PFC and striatum, the observed pattern is the opposite. Prioritized DEGs were mostly upregulated in PFC (1.77 and 0.5, respectively), while they were mostly downregulated in striatum (−2.25 and −1.20, respectively). These results indicate that DEG and the genes functionally relevant in enriched terms are differentially regulated in PFC than in striatum.

The chord plots for KEGG (Figure S2 and S3) and GO (Figures S4 and S5) enriched terms allowed the analysis of the number of terms associated with each functional candidate gene. It was possible to note that while some genes were associated with several enriched terms (e.g., *Pink1*, *Bdnf*, *Gria1*), other genes were associated with just one or few terms (e.g., *Il1rap*, *Scn1a*, *Cep97*).

### 3.3 Potential transcription factors

**Figure 3:**
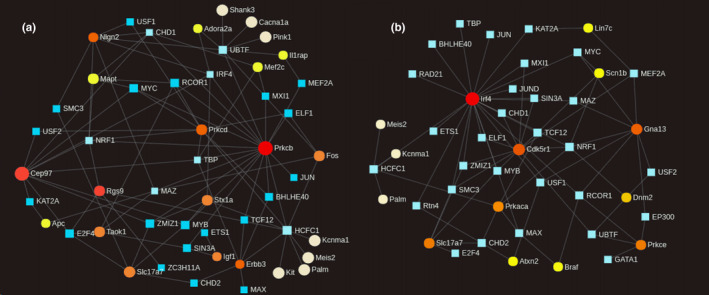
TF‐target gene network for the prioritized genes identified in the PFC (a) and striatum (b). The blue squares represent the potential transcription factors (TFs), and the circles, the prioritized genes. Each edge between a TF and a gene represents a potential regulatory activity. The colors of the circles, as well as the area of the circle, represent the number of possible TFs associated with this gene. The darker the red colors of the circle, the larger the number of TFs associated with it

**Figure 4:**
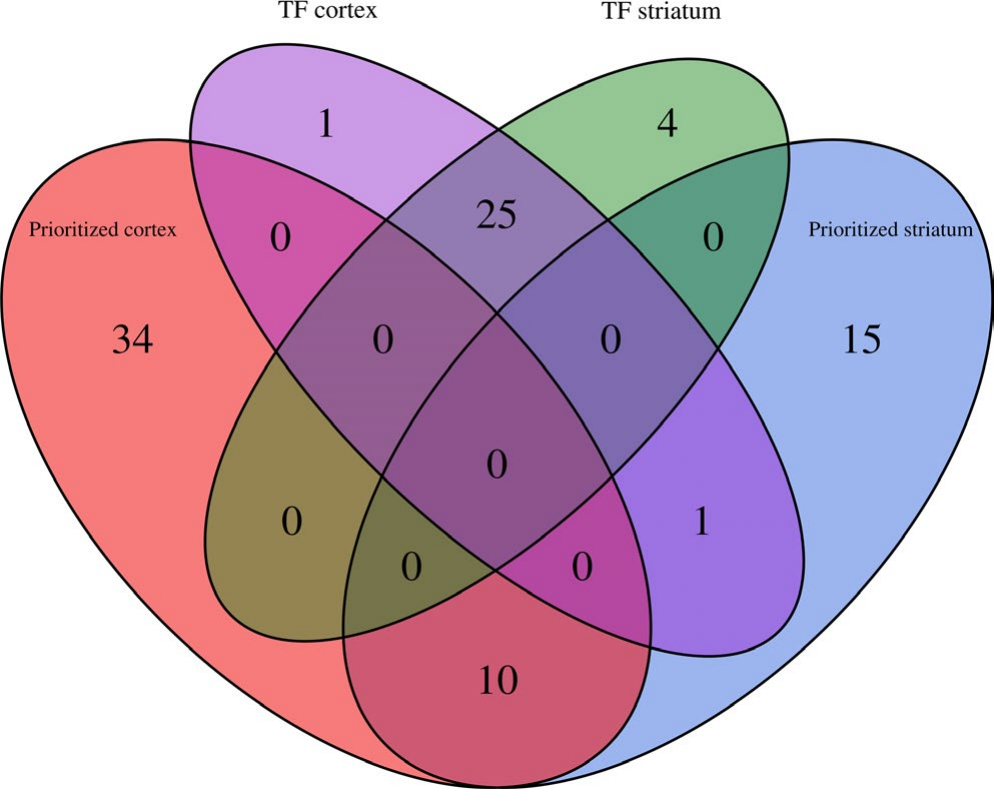
Venn diagram representing the sharing pattern among the prioritized genes. PFC (red ellipse), striatum (blue ellipse), and the potential transcription factors (TFs) in PFC (purple ellipse) and striatum (green ellipse)

**Figure 5:**
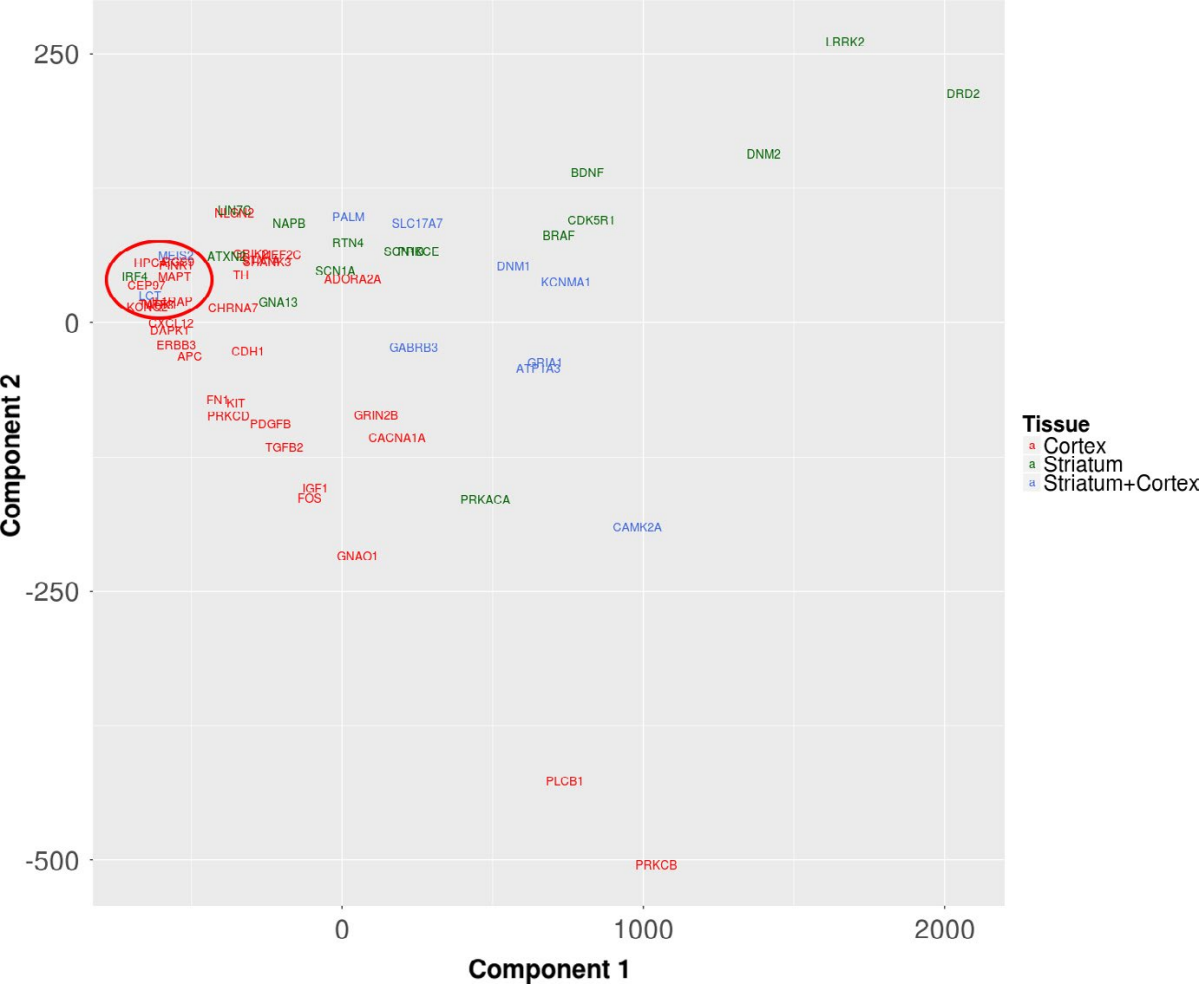
Multidimensional scaling plot (MDS) clustering the prioritized genes identified in the PFC (red symbols), striatum (green symbols), and both tissues (blue symbols) based on the functional annotation. The genes were clustered based on the Euclidian distance obtained from the hamming distance for the incidence matrix composed by genes and the most functionally relevant enriched GO and KEGG terms. The red circle highlights the position of the *IRF4* gene

Figure 3 depicts the TF‐target gene network for PFC (3A) and striatum (3B). Each node in this network represents a gene (circles) or a TF (squares), and each edge between two nodes represents evidence of regulatory interaction. Table 3 shows the 10 TFs with the highest centrality metric in each network, as well as the potential target genes. The centrality metrics for all the nodes presented in Figure 4 are listed Table S7. The interferon regulatory factor gene (*Irf4*), prioritized in the striatum, was identified as one of the TFs with the highest centrality metric in the PFC network (Figure 5).

**Table 3:** Top 10 transcription factors (TFs) with the highest centrality metrics in prefrontal cortex (PFC) and striatum

To 10 TF for centrality metric			
Prefrontal Cortex		Striatum	
Gene symbol	Degree/ Betweenness	Gene symbol	Degree/ Betweenness
*Hcfc1*	14/272.37	*Nrf1*	6/93.25
*Ubtf*	9/199.78	*Hcfc1*	5/127.11
*Nrf1*	8/75.07	*Rcor1*	4/91.13
*Mas*	8/72.63	*Tcf12*	4/25.43
*Tbp*	7/60.71	*Chd2*	4/20.57
*Irf4*	7/52.27	*Usf1*	3/43.51
*Chd1*	5/43.89	*Zmiz1*	3/26.47
*Myb*	5/33.49	*Smc3*	3/26.47
*Zmiz1*	5/33.49	*Myb*	3/26.47
*E2f4*	5/33.15	*Ubtf*	3/25.39

The PCA plot in Figure 5 was created using the first two principal components of a multidimensional scaling (MDS) analysis. The components were obtained using the Euclidean distance between each pair of genes in the dataset. The Euclidian distance was estimated from a nongeometric distance (Hamming distance) in order to avoid geometric approximations. In summary, the MDS analysis is the final step for the functional similarity analysis among the genes. After some transformations the incidence matrix composed by the DEG and enriched terms (BP, MF, CC, and KEGG) are represented in a two‐dimensional map. The first and second components explain 79.77% and 6.93% of the variance, respectively. Together, both components explain more than 86% of the total variance on the difference between genes, regarding the functional profile. In sum, Figure 5 reflects the results of a functional clustering analysis performed using the all the GO terms associated (filtering was not applied based on p‐value) with the prioritized genes. Additionally, it was possible to identify interesting clustering patterns from the PCA analysis. Most part of the PFC genes are mapped in the negative side of the first component (explaining 79.77% of the total variance) and in the positive side of the second component (explaining 6.93% of the total variance). On the other hand, most of the striatum genes are mapped in the opposite coordinates, positive side of the first component, and positive side of the second component. Interestingly, genes expressed in both structures clustered in intermediary areas in the PCA. Despite the PCA does not use the expression values to perform the cluster analysis (the functional annotation was used as input for this analysis), the results suggest a possible specialization of the genes expressed in each region. Figure 5 indicates that *Irf4* has a more similar functional pattern than the PFC‐prioritized genes (red circle). In addition, the cluster analysis (Figure 5) showed that in the striatum *Dnm2*, *Lrrk2,* and *Drd2* are the genes with the largest weight in the first, which explains around 80% of variance, and second components (along with BDNF). Furthermore, these striatal genes along with *Plcb1* and *Prkcb,* in PFC, appeared detached from the other genes within and between the tissues, suggestive of a tissue‐specific functional pattern.

### 3.4 Postmortem human brain qPCR results

Table 4 summarizes the demographic and clinical characteristics of AUD and control subjects. Compared to controls, the AUD subjects had higher BMI, daily alcohol intake, drinks per week, blood alcohol concentration (BAC) at time of death, higher pack‐years cigarettes, and younger drinking initiation, but they did not differ in age. Moreover, AUD subjects had a lower brain weight and smaller brain volumes than controls.

**Table 4:** Demographic and clinical characteristics of alcohol use disorder (AUD) and control subjects. BMI = body mass index; PMI = postmortem interval (hour); BAC = blood alcohol concentration (g/100ml) at death

Characteristics	AUD (*n* = 10)	Controls (*n* = 13)	*p*‐value
Age	50.55 ± 6.07	49.94 ± 11.32	*p* > .05
BMI	24.64 ± 5.40	33 ± 1.46	*p* = .023
PMI	38.91 ± 12.69	31.06 ± 13.94	*p* > .05
Brain Weight	1,387.73 ± 127.71	1506.63 ± 106.81	*p* = .015
Age onset drinking	18.55 ± 4.13	24 ± 4.86	*p* = .007
BAC	0.197 ± 0.14	0.002 ± 0.008	*p* = .0001
Drinking (g/day)	233.27 ± 118.09	18.51 ± 19.87	*p* = .0001
Drinks per week	125.36 ± 89.99	9.81 ± 9.60	*p* = .0001
Pack‐years cigarettes	45.09 ± 19.22	4.07 ± 13.87	*p* = .0001

Exploratory correlations between mRNA levels and drinking, smoking, and demographics (age and BMI) for the AUD group and controls are shown in Tables S8 and S9. The correlation analyses showed a positive correlation between the levels of *IRF4* in the NAc with BAC (*r* = .670, *p* = .034) and pack‐years cigarettes (*r* = .611, *p* = .046) just in the control groups. For the AUD group, positive correlations were observed between the DNM2 mRNA levels in NAc and BAC (*r* = .721, *p* = .043), and between the PLCB1 levels in PFC and daily alcohol intake (*r* = .641, *p* = .046). Correlations between demographic (age and BMI) data and mRNA levels of all genes evaluated in NAc and PFC were not significant in both AUD and control groups.

**Figure 6:**
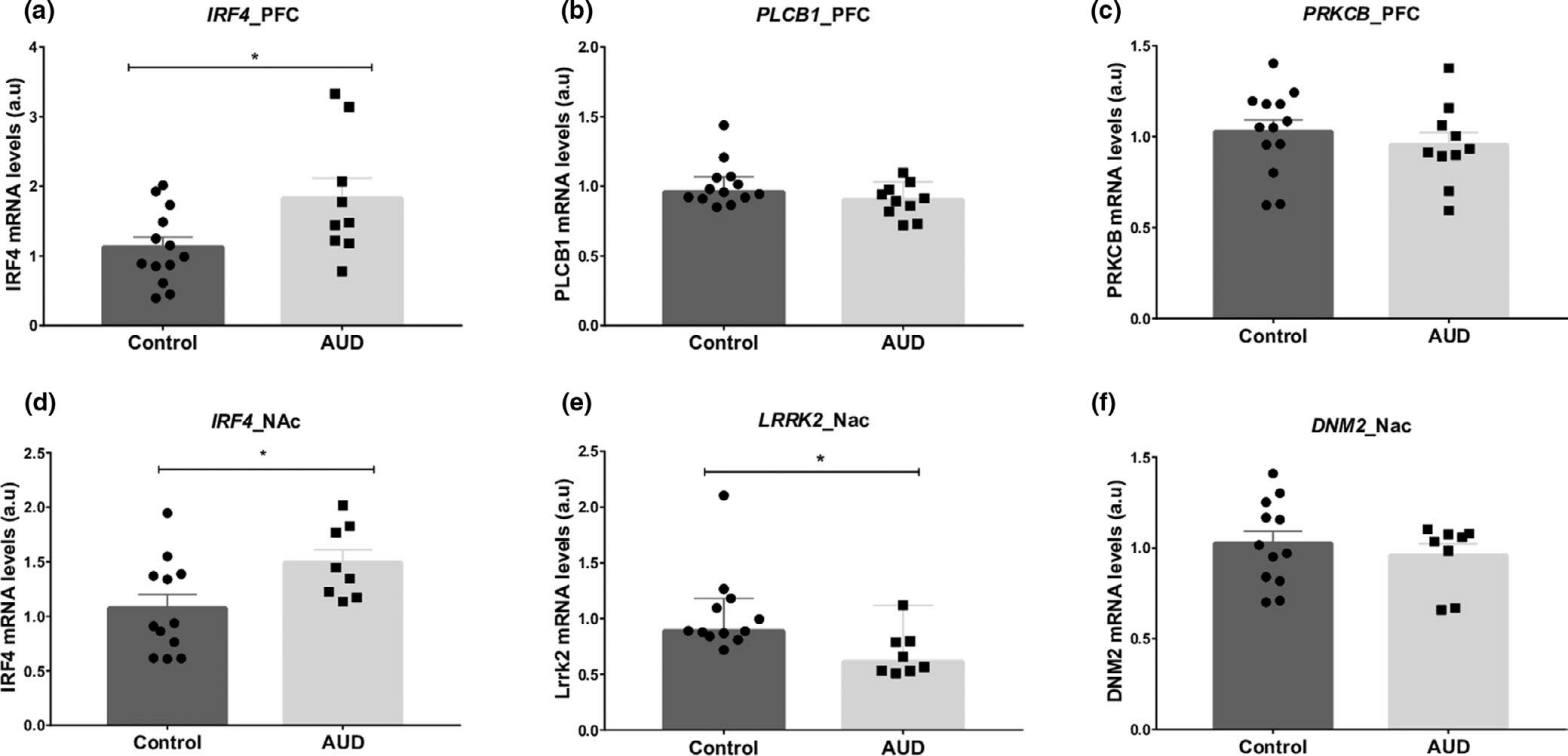
Relative mRNA quantification in postmortem brain tissue from alcohol use disorder (AUD) and control subjects. Prefrontal cortex (PFC) and nucleus accumbens (NAc). Relative mRNA levels of (a) *IRF4*, (b) *PLCB1*, (c) *PRKCB,* (d) *IRF4,* (e) *LRRK2,* and (f) *DNM2*. In a, d, and e, *^#^ *p* < .05 different from control. IFR4 in NAC (*p* = .034, q = 0.066) and PFC (*p* = .030, q = 0.066) and LRRK2 (*p* = .005, q = 0.030) in NAc, survived the FDR 5% correction. Unpaired *t* test was used to analyze the differences between the groups for IRF4 and DNM2 in NAc and for IRF4 and PRKCB in PFC. Mann–Whitney was used to analyze LRRK2 in NAc and PLCB1 in PFC. Results are presented as mean ± *SEM* for (a, c, d, and f) and median with 95% CI for (b and e)

The cluster analysis for the prioritized genes (Figure 5) suggested a tissue‐specific functional pattern for *Irf4*, *Dnm2, Lrrk2, Prkcb,* and *Plcb1* genes in the context of compulsive ethanol drinking. Postmortem human brain from individuals with AUD was used to test whether those prioritized genes in our animal model that present face validity for human alcohol addiction would also be found dysregulated in humans with AUD. Therefore, we analyzed their transcriptional regulation in the PFC and NAc. The q values represent the p‐value correct by the FDR 5%. No differences were observed for *PLCB1* (U = 38, *p* = .101, q = 0.121) and *PRKCB* (t = 0.776, *df* = 21, *p* = .445, *p* = .066) in PFC (Figure 6b and 6c), or for *DNM2* (t = 0.674, *df* = 18, *p* = .508, q = 0.508) in NAc (Figure 6f). For *IRF4* in PFC, ROUT method identified one outlier in the AUD group. *IRF4* was upregulated in both PFC (t = 2.33, *df* = 20, *p* = .030, q = 0.066), and NAc (t = 2.292, *df* = 18, *p* = .034, q = 0.066) in AUD subjects when compared with controls (Figure 6a,d). For *LRRK2*, ROUT method identified one outlier in the control group. *LRRK2* was downregulated (U = 11, *p* = .005, q = 0.030) in NAc of AUD compared to controls (Figure 6e). Just *LRRK2* in NAc was significant after the FDR 5% correction.

## 4 DISCUSSION

In the present study, using a guilt‐by‐association approach in microarray data from an animal model of inflexible ethanol consumption (da Silva E Ribeiro et al., 2012; Silva et al., 2016), we prioritized 44 DEGs in PFC and 26 in striatum. Among those genes, the *Irf4* and *Lrrk2* in addition to presenting a tissue‐specific pattern of regulation in the inflexible drinker mice were also differentially regulated in the PFC and NAc of postmortem brain from AUD subjects. These results suggest a crucial role for *Irf4* and *Lrrk2* in the context of compulsive ethanol intake in mice and humans.

The guilt‐by‐association heuristic has led to the identification of genes that are believed to be associated with a specific disease, phenotype, or common cellular function. Although the guilt‐by‐association approach is widely applied in studies aiming to scrutinize the biological processes associated with complex traits (Albert & Lemonde, 2004; Altshuler et al., 2000; Bowcock, 2007; Guo et al., 2013; Stuckenholz et al., 1999; Ziganshin & Elefteriades, 2016), the combination of GUILDify and ToppGene in a single analysis is a new approach in the literature regarding functional prioritization (Pas et al., 2018; Kominakis et al., 2017).

The selection of terms and biological processes to build the trained list, using the GUILDify, can be considered a biased approach. However, this bias is consciously introduced in the analysis due to the functional relevance of the processes to the target phenotype. In our specific case, our phenotype is the inflexible pattern of ethanol intake that includes characteristics such as long‐term high ethanol intake, heightened anxiety during withdrawal, and persistent intake despite ethanol adulteration with quinine. Those behaviors can be resultants both from pre‐existing genetic differences and from persistent changes in neuronal process induced by ethanol that are already described in the literature and can be represented by the keywords chosen here (e.g., "Firing midbrain dopamine"; "Long ‐term potentiation"; "Inhibition NMDA"). The ToppGene will not use these keywords to select our genes; instead, the software uses the similarities between the functional patterns of the genes presented in the candidate gene list and the trained gene list. Therefore, the prioritized genes presented in this study can be interpreted as a statistical measure of how much the functional profile of each candidate gene is similar with the whole functional profile of the trained list (GUILDify) that reflects the process behind the alcohol addiction. Consequently, even if some of the genes in our initial list of candidate genes were not previously assigned to our selected terms, we were able to identify a possible function of these genes in our candidate processes due to the functional similarity. However, it is important to highlight that it is not our goal, and neither is possible to detect all genes that are associated with the inflexible pattern of ethanol intake. Our goal is to find and select genes with higher evidence of association with the process that are crucial to the development and maintenance of the inflexible phenotype observed in mice.

Among the prioritized genes in PFC and striatum, we observed several direct and indirect molecular targets for ethanol. The direct targets comprised *Drd2*, *Gria1*, *Grik2*, *Grin2b*, *Chrna7,* and *Gabrb3,* and the protein products of these genes are affected by acute and chronic ethanol (Abrahao et al., 2017). The indirect targets comprised genes encoding proteins for which there is no evidence of an ethanol‐binding site but that are affected by chronic ethanol exposures and included intracellular signaling proteins (*Plcb1*, *Rgs,9* and *Prkcb* in PFC and *Prkaca* in striatum), proteins involved in endocytosis and vesicle trafficking (*Dnm2*, *Atxn2,* and *Napb* in striatum), and transcription factors (*Meis2* and *Tgfb2* in PFC and *Irf4* in striatum) (Abrahao et al., 2017). These are well‐defined genes in the context of ethanol, and their prioritization in our animal model shows the validity of our methodology.

The cluster analysis (Figure 5) revealed that some of the prioritized genes presented a specific functional pattern in each tissue analyzed. The *Plcb1* and *Prkcb* genes appear detached from the other genes in the PFC just as *Lrrk2*, *Drd2,* and *Dnm2* genes appeared detached from other genes in the striatum. Collectively, those genes play roles that ultimately contribute to synaptic plasticity, regulating behavioral outcomes associated with specific neural circuits. Additionally, they exhibit a close relationship in which their products activate each other (*Plcb1* and *Prkcb)* or participate in the same signaling pathway (*Lrrk2* and *Dnm2*), indicating an orchestrated network. Moreover, the inverse pattern of regulation assessed by the z‐score calculation showed that most of the prioritized genes in the PFC are upregulated while in the striatum they are downregulated. Additionally, the prioritized genes in the PFC and striatum are differently regulated in comparison with all DEGs found in the same tissue, highlighting that prioritized genes are working in distinct ways in response to chronic alcohol. Unfortunately, our study could not determine causal interactions between brain regions; thus, further studies are necessary to elucidate the regulatory role that striatum pursue over PFC or vice versa in response to alcohol intake.

The transcription factor analysis showed a pattern for *Irf4* suggestive of a possible regulation of genes in the PFC over the striatum or vice versa. This gene belongs to the interferon regulatory factor (IRF) family of TFs related to gene expression regulation and immune response activation (Negishi et al., 2017). Despite our finding that *Irf4* was prioritized in the striatum, it was a TF with the highest centrality metric in the PFC. Furthermore, this gene appeared in the cluster analysis together with genes in the PFC and showed a more similar functional pattern with this tissue. This result suggests that *Irf4* may play a crucial role in the opposite pattern of regulation observed between PFC and striatum. The activation of TFs and the neuroimmune responses are two crucial mechanisms of the brain in response to chronic ethanol and can trigger longer‐term molecular neuroadaptations (Koob & Volkow, 2016). In the TF‐target gene network, the *Irf4* in the striatum is also associated with diverse TFs such as T*bp, Elf1, Mxl1, Jun, Zmiz1, and Chd*. So far, studies have only reported on the role of the IRF family in inflammation and secondary diseases from chronic alcohol (Petrasek et al., 2011; Seki & Brenner, 2008). Therefore, the association found here highlights the *Irf4* as an important target to be investigated in animal models of alcohol intake.

To investigate whether the genes that showed a tissue‐specific pattern of regulation in the inflexible drinker animals (*Irf4, Plcb1, Prkcb, Dnm2,* and *Lrrk2*) were also differentially regulated in AUD, we performed a quantitative PCR analysis in PFC and NAc of postmortem brains of AUD subjects. Although we did not observe any differences in the transcriptional regulation of *PLCB1, PRKCB,* and *DNM2,* we observed an upregulation of *IRF4* in both PFC and NAc of humans (these effects did not survive FDR 5% correction). Nevertheless, this uncorrected result corroborates our hypothesis of *Irf4* role in the control of transcriptional regulation and activity in PFC and striatum in inflexible drinking mice and highlights its role in the compulsive ethanol drinking. Since the finding for *IRF4* in AUD was significant only at an uncorrected level, we consider them preliminary and in need of replication.

We also observed that *LRRK2* was significantly downregulated in the NAc of humans with AUD. We had previously suggested a role of *Lrrk2* in the transition to the loss of control over voluntary ethanol intake; however, this finding differs from the upregulation of this gene found in the striatum (dorsal and ventral) of inflexible drinker mice (da Silva E Silva et al., 2016). Interestingly, our recent work on a ethanol preference behavior in a zebrafish model also showed a upregulation of *lrrk2* in the brain of animals with inflexible phenotype and demonstrates the role of *lrrk2* in driving the preference for ethanol, since the treatment with its inhibitor (GNE‐0877) reduced the ethanol preference in the inflexible group (“Inhibition of Lrrk2 reduces ethanol preference in a model of acute exposure in zebrafish,” Paiva et al., 2020). Though these transcriptional differences could reflect distinct responses between the NAc and the dorsal striatum or between species, it is also possible that it is not either the up‐ or downregulation of this gene, but it is dysregulation in general, that is relevant to the loss of control over ethanol intake.

In conclusion, the present study is the first one in the alcohol field to apply the guilt‐by‐association approach using the GUILDify and ToppGene to prioritize genes. We generate a list of DEG in both PFC and striatum that we do believe to be implicated in the transition of normal to compulsive ethanol intake and that can be tested in future functional studies. Most of the prioritized genes are involved in the establishment of synapse plasticity, a crucial process that leads to neuroadaptations and ethanol‐related behaviors. The test of some of the prioritized genes that showed a tissue‐specific pattern in postmortem brain tissue allowed us to uncover evidence from both human AUD and inflexible drinker animals for *Ifr4* underlying the pattern of regulation observed between the PFC and striatum. Our results also highlight a prominent role of *LRRK2* in the pattern of responses to compulsive alcohol drinking in humans and mice.

## CONFLICT OF INTEREST

No potential conflict of interest was reported by the authors.

## AUTHOR CONTRIBUTION

LMC, PASF, and ALBG were responsible for the in silico study concept and design. DSS contributed to the acquisition of microarray data. PASF performed the bioinformatic analysis. LMC and PASF assisted with data analysis and interpretation of findings. LMC drafted the manuscript. ALBG, CW, NDV, PASF, IMP, *SD*, and ASP provided critical revision of the manuscript for important intellectual content. CW and NDV provided the postmortem brain tissue from humans with alcohol use disorder. All authors critically reviewed content and approved the final version for publication.

### Peer Review

The peer review history for this article is available at https://publons.com/publon/10.1002/brb3.1879.

## Supporting information

Fig S1

Fig S2

Fig S3

Fig S4

Fig S5

Table S1

Table S2

Table S3

Table S4

Table S5

Table S6

Table S7

Table S8

Table S9
