# Supplementary figures and images for: Identifying functionally relevant candidate genes for inflexible ethanol intake in mice and humans using a guilt‐by‐association approach
Source: Brain Behav. 2020 Oct 23;10(12):e01879. doi: 10.1002/brb3.1879 (PMC7749619; doi:10.1002/brb3.1879)

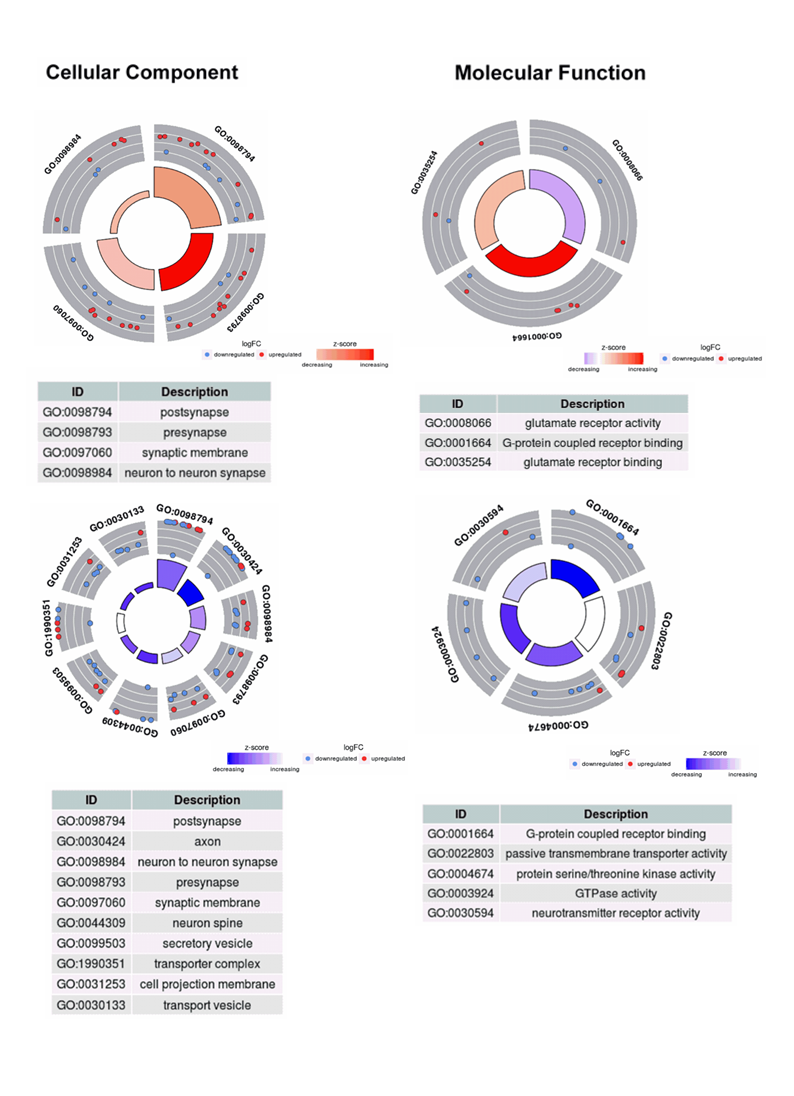

Supplement: Supplementary file 1 — Fig S1 [file BRB3-10-e01879-s001.tiff]

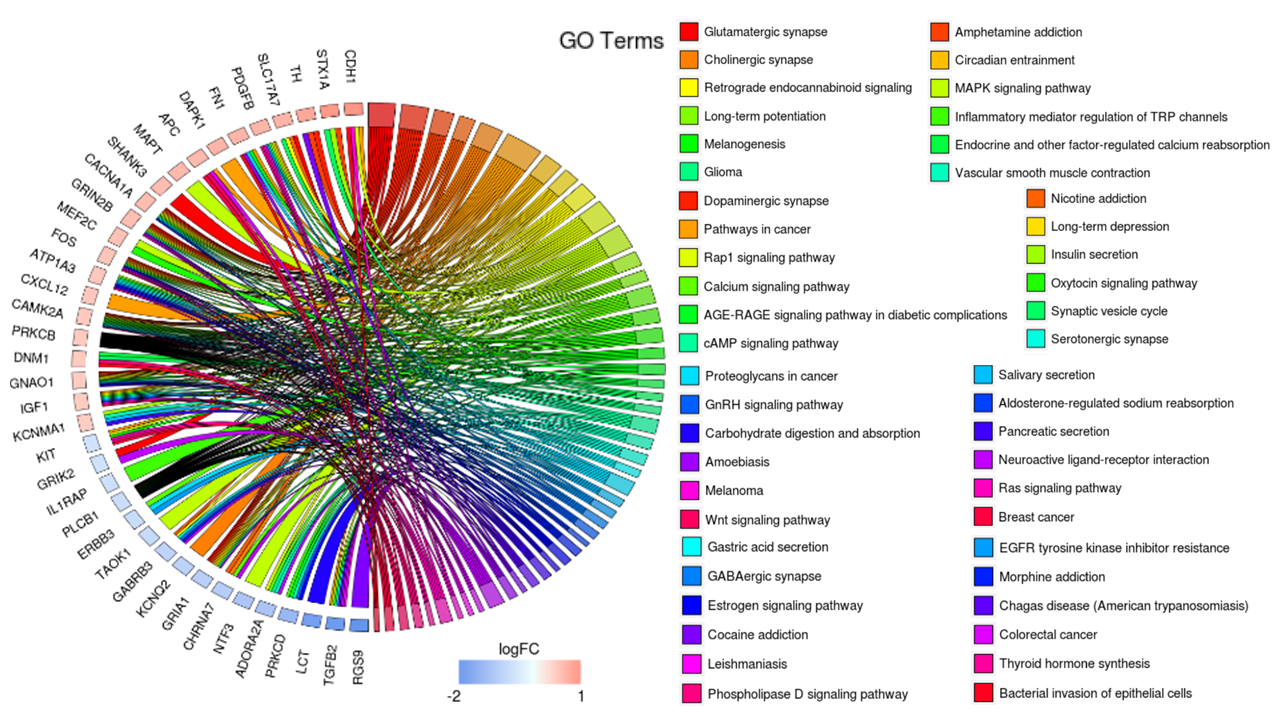

Supplement: Supplementary file 2 — Fig S2 [file BRB3-10-e01879-s002.tiff]

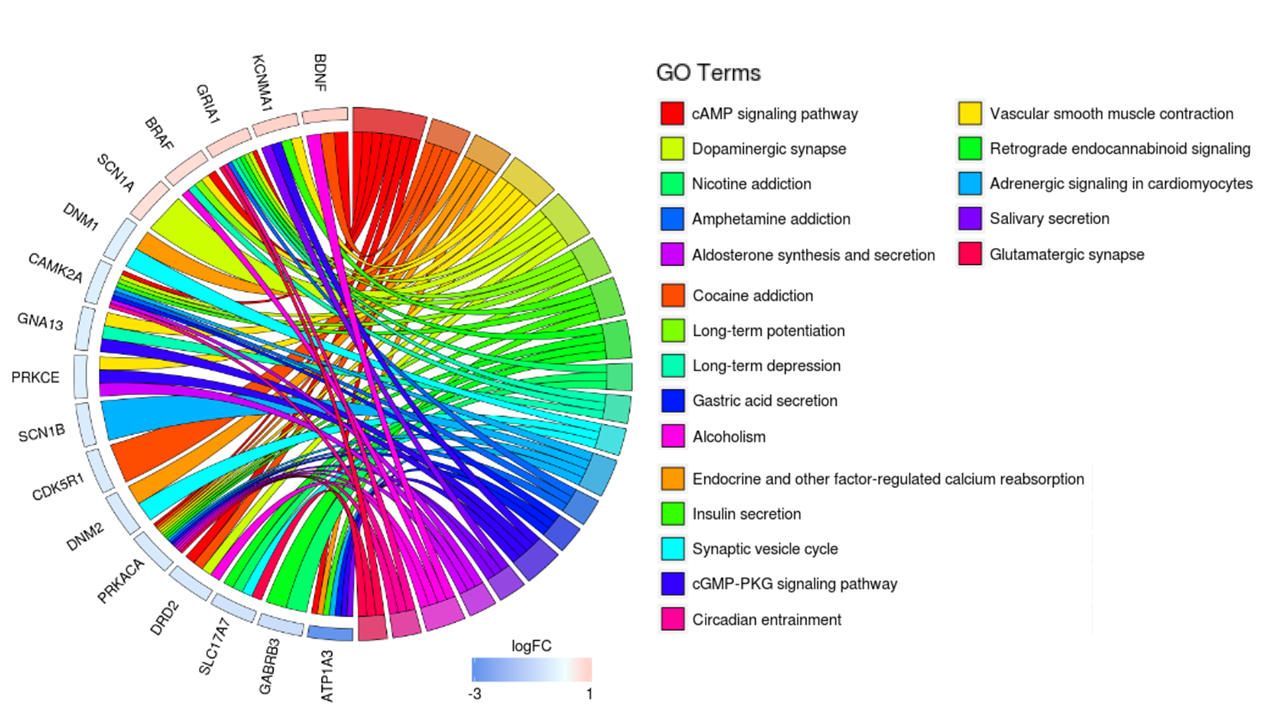

Supplement: Supplementary file 3 — Fig S3 [file BRB3-10-e01879-s003.tiff]

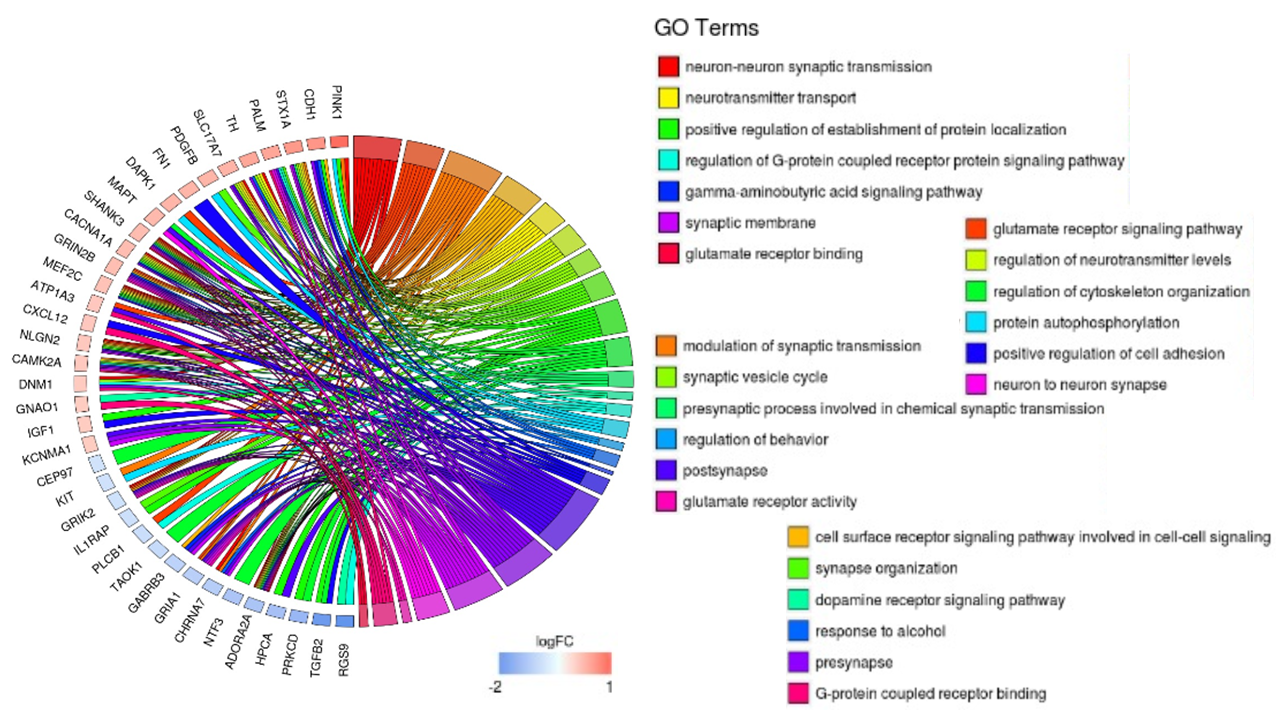

Supplement: Supplementary file 4 — Fig S4 [file BRB3-10-e01879-s004.tiff]

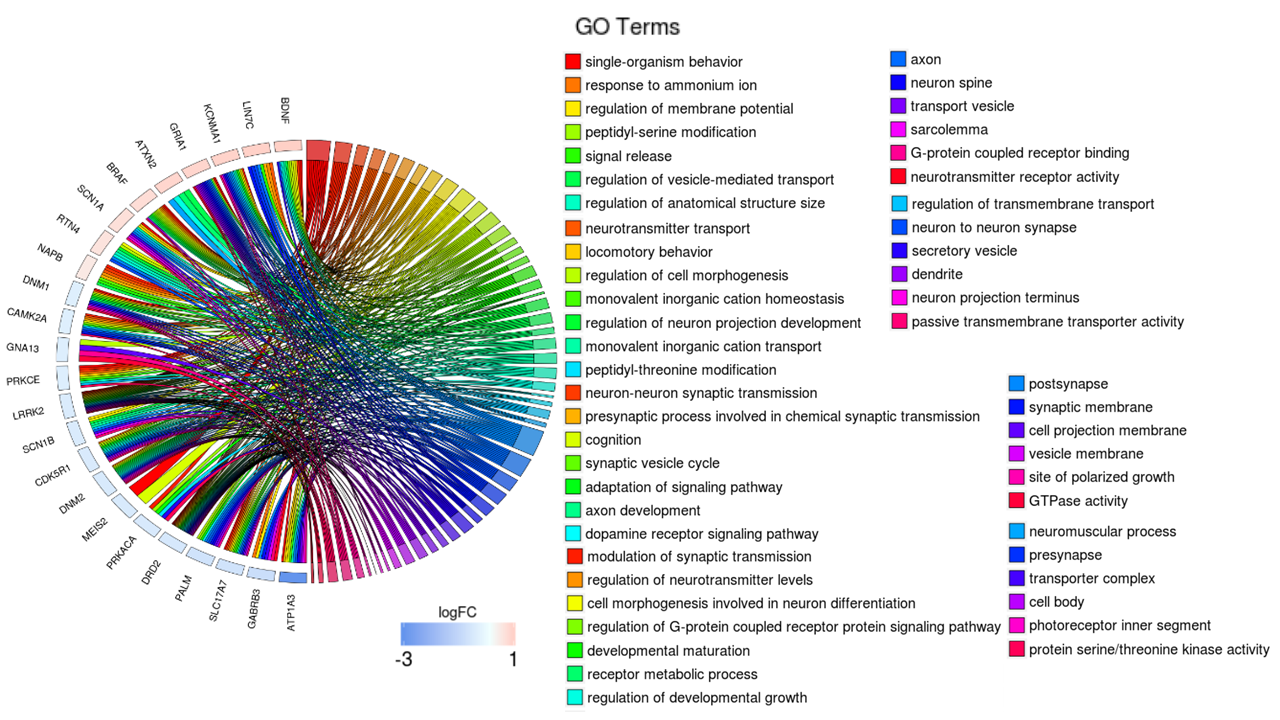

Supplement: Supplementary file 5 — Fig S5 [file BRB3-10-e01879-s005.tiff]
