# Supplementary material for: Identifying functionally relevant candidate genes for inflexible ethanol intake in mice and humans using a guilt‐by‐association approach
Source: Brain Behav. 2020 Oct 23;10(12):e01879. doi: 10.1002/brb3.1879 (PMC7749619; doi:10.1002/brb3.1879)
Supplement: Supplementary file 13 — Table S8 [file BRB3-10-e01879-s013.docx]

|  | **Age** | **BMI** | **Age Onset drinking** | **BAC (g/100ml)** | **Daily Alcohol Intake (g)** | **Drinks per week** | **Pack-year Cigarrettes** | **Lrrk2 mRNA (Nac)** | **Dnm2 mRNA (Nac)** | **Irf4 mRNA (Nac)** | **Prkcb mRNA (PFC)** | **Plcb1 mRNA (PFC)** | **Irf4 mRNA (PFC)** |
| --- | --- | --- | --- | --- | --- | --- | --- | --- | --- | --- | --- | --- | --- |
| **Age** | 1 |  |  |  |  |  |  |  |  |  |  |  |  |
| **BMI** | -0.541* | 1 |  |  |  |  |  |  |  |  |  |  |  |
| **Age onset drinking** | 0.033 | 0.138 | 1 |  |  |  |  |  |  |  |  |  |  |
| **BAC (g/100ml)** | -0.015 | 0.073 | 0.024 | 1 |  |  |  |  |  |  |  |  |  |
| **Daily Alcohol Intake (g)** | 0.34 | 0.148 | -0.03 | -0.17 | 1 |  |  |  |  |  |  |  |  |
| **Drinks per week** | 0.384 | -0.018 | 0.148 | -0.052 | .943** | 1 |  |  |  |  |  |  |  |
| **Pack-year cigarretts** | -0.078 | 0.252 | 0.045 | .963** | -0.124 | -0.088 | 1 |  |  |  |  |  |  |
| **Lrrk2 mRNA (Nac)** | 0.215 | -0.34 | -0.187 | 0.183 | 0.492 | 0.375 | 0.168 | 1 |  |  |  |  |  |
| **Dnm2 mRNA (Nac)** | -0.117 | 0.221 | 0.464 | 0.413 | 0.236 | 0.408 | 0.319 | 0.161 | 1 |  |  |  |  |
| **Irf4 mRNA (Nac)** | 0.077 | 0.043 | 0.357 | .670* | 0.161 | 0.253 | .611* | 0.518 | .768** | 1 |  |  |  |
| **Prkcb mRNA (PFC)** | 0.073 | -0.231 | -0.324 | 0.281 | 0.201 | 0.294 | 0.247 | 0.094 | -0.147 | -0.228 | 1 |  |  |
| **Plcb1 mRNA (PFC)** | -0.03 | 0.391 | -0.001 | -0.116 | -0.152 | -0.262 | -0.02 | -0.2 | -0.382 | -0.251 | -0.29 | 1 |  |
| **Irf4 mRNA (PFC)** | 0.386 | 0.177 | 0.33 | -0.108 | -0.132 | -0.117 | -0.119 | -0.293 | 0.058 | 0.131 | -.686** | 0.401 | 1 |

**Supplementary Table 8.** Zero-order correlations between relative mRNA levels and demographic and clinical variables in Control group. ** Correlation is significant at the 0.01 level (2-tailed). * Correlation is significant at the 0.05 level (2-tailed).
