# Supplementary material for: Identifying functionally relevant candidate genes for inflexible ethanol intake in mice and humans using a guilt‐by‐association approach
Source: Brain Behav. 2020 Oct 23;10(12):e01879. doi: 10.1002/brb3.1879 (PMC7749619; doi:10.1002/brb3.1879)
Supplement: Supplementary file 14 — Table S9 [file BRB3-10-e01879-s014.docx]

**Table S9.** Zero-order correlations between relative mRNA levels and demographic and clinical variables in Alcohol Use disorder (AUD). ** Correlation is significant at the 0.01 level (2-tailed). * Correlation is significant at the 0.05 level (2-tailed).

|  | **Age** | **BMI** | **Age Onset drinking** | **BAC (g/100ml)** | **Daily Alcohol Intake (g)** | **Drinks per week** | **Pack-year Cigarrettes** | **Lrrk2 mRNA (Nac)** | **Dnm2 mRNA (Nac)** | **Irf4 mRNA (Nac)** | **Prkcb mRNA (PFC)** | **Plcb1 mRNA (PFC)** | **Irf4 mRNA (PFC)** |
| --- | --- | --- | --- | --- | --- | --- | --- | --- | --- | --- | --- | --- | --- |
| **Age** | 1 |  |  |  |  |  |  |  |  |  |  |  |  |
| **BMI** | -0.213 | 1 |  |  |  |  |  |  |  |  |  |  |  |
| **Age onset drinking** | 0.39 | -0.102 | 1 |  |  |  |  |  |  |  |  |  |  |
| **BAC (g/100ml)** | 0.282 | 0.322 | 0.212 | 1 |  |  |  |  |  |  |  |  |  |
| **Daily Alcohol**  **Intake (g)** | 0.052 | .698* | 0.072 | 0.49 | 1 |  |  |  |  |  |  |  |  |
| **Drinks per week** | 0.048 | .720* | -0.069 | 0.327 | .968** | 1 |  |  |  |  |  |  |  |
| **Pack-year**  **cigarretts** | .850** | -0.231 | 0.257 | 0 | -0.009 | 0.038 | 1 |  |  |  |  |  |  |
| **Lrrk2 mRNA (Nac)** | -0.206 | 0.551 | -0.164 | -0.386 | -0.425 | -0.353 | 0.041 | 1 |  |  |  |  |  |
| **Dnm2 mRNA (Nac)** | 0.415 | -0.517 | 0.297 | -.721* | -0.489 | -0.438 | 0.309 | 0.11 | 1 |  |  |  |  |
| **Irf4 mRNA (Nac)** | -0.064 | 0.214 | -0.098 | 0.125 | -0.4 | -0.449 | -0.391 | -0.054 | 0.065 | 1 |  |  |  |
| **Prkcb mRNA (PFC)** | -0.121 | 0.319 | -0.348 | -0.152 | -0.324 | -0.285 | 0.234 | .787* | -0.203 | -0.359 | 1 |  |  |
| **Plcb1 mRNA (PFC)** | 0.077 | -0.105 | -0.299 | -0.524 | -.641* | -0.555 | 0.197 | 0.361 | 0.254 | -0.092 | 0.21 | 1 |  |
| **Irf4 mRNA (PFC)** | -0.179 | 0.158 | -0.513 | -0.342 | -0.405 | -0.165 | 0.095 | 0.44 | -0.422 | 0.431 | 0.309 | 0.535 | 1 |
